# Quantum criticality of excitonic Mott metal-insulator transitions in black phosphorus

**DOI:** 10.1038/s41467-022-35567-w

**Published:** 2022-12-17

**Authors:** Binjie Zheng, Junzhuan Wang, Qianghua Wang, Xin Su, Tianye Huang, Songlin Li, Fengqiu Wang, Yi Shi, Xiaomu Wang

**Affiliations:** 1 School of Electronic Science and Engineering, Nanjing University, 210093 Nanjing, China; 2 School of Physics, Nanjing University, 210093 Nanjing, China

**Keywords:** Phase transitions and critical phenomena, Two-dimensional materials, Optical properties and devices

## Abstract

Quantum phase transition refers to the abrupt change of ground states of many-body systems driven by quantum fluctuations. It hosts various intriguing exotic states around its quantum critical points approaching zero temperature. Here we report the spectroscopic and transport evidences of quantum critical phenomena of an exciton Mott metal-insulator-transition in black phosphorus. Continuously tuning the interplay of electron-hole pairs by photo-excitation and using Fourier-transform photo-current spectroscopy as a probe, we measure a comprehensive phase diagram of electron-hole states in temperature and electron-hole pair density parameter space. We characterize an evolution from optical insulator with sharp excitonic transition to metallic electron-hole plasma phases featured by broad absorption and population inversion. We also observe strange metal behavior that resistivity is linear in temperature near the Mott transition boundaries. Our results exemplify an ideal platform to investigating strongly-correlated physics in semiconductors, such as crossover between superconductivity and superfluity of exciton condensation.

## Abstract (web summary)

The exciton Mott transition refers to a transition from an insulating state of gas-like excitons to strongly correlated electron-hole plasma phases in photoexcited semiconductors. Here the authors experimentally study such a transition in black phosphorus and reveal its quantum critical properties.

## Introduction

Bound electron–hole pairs, known as excitons, determine the photo-responses of semiconductors. At extreme conditions, the gas-like excitons dissociate into strongly correlated electron–hole plasma phases. In this so-called exciton Mott transition, the optical properties of a semiconductor sharply change with the screening modulated Coulomb correlation effect^1–3^. It is believed to closely relate to exotic matter states, such as superfluidity, superconductivity, and non-Fermi liquid of electron–hole pairs. In conventional bulk materials, the electron–hole phase transition is driven by intense optical excitation and at cryogenic conditions^4–8^. These extreme conditions limit practical applications; and more importantly, the detailed phase transition process and the associated evolution of quantum states remain elusive for the same reason.

Black phosphorus (BP), an emerging two-dimensional (2D) semiconductor, exhibits a unique anisotropic band structure and optical tunability^9^. Its highly anisotropic optical responses reduced dielectric screening and enhanced Coulomb interactions in the 2D regime directly result in strong many-body interaction and correlation and thus significantly lower the Mott density. Therefore, it potentially enables comprehensive characterization and flexible manipulation of exciton Mott transition^10–15^. Here we systemically investigate the excitonic metal–insulator transition in BP film by a combination of optical spectroscopy and electrical transport measurement.

## Results

### Electron–hole phases in BP

**Fig. 1:**
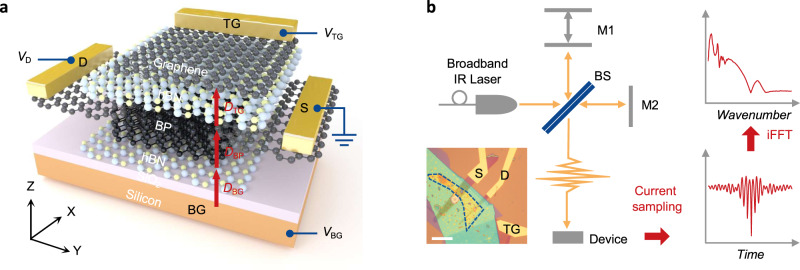
Device configuration and measurement scheme. **a** Schematic view of a typical dual-gate BP transistor. Top and bottom gate voltages (*V*_TG_ and *V*_BG_, respectively) are applied to control the carrier density and electric displacement field in the sample (*D*_BP_). **b** Illustration of the interferometer setup where M1, M2, and BS represent the movable mirror, stationary mirror, and beam splitter, respectively. Photocurrent spectrum (top right) which is obtained from inverse Fourier-transform of photocurrent interferogram (bottom right). Inset: optical micrograph of a BP (dashed line) device. Scale bar, 20 μm.

Figure 1a schematically shows the dual-gate BP device used in our study. A BP thin film ~10 nm was encapsulated between two hexagonal boron nitride (hBN) flakes. The two hBN flakes, together with the glove-box-based fabrication process, ensure BP is of high quality. To keep the flatness of the whole structure, we also used few-layer graphene flakes to form source, drain, and top gate contacts. The dual-gate structure allows applying an electric displacement field with constant doping in photocurrent and transport measurements.

Previously, photo-emission spectra were widely used to study the non-equilibrium electron–hole plasma (EHP) phenomenon under ultra-fast excitation. Unfortunately, these spectra less manifest the rich information from non-radiative features of the whole many-body band-structure, along as any transport attributes. We thus employed a Fourier transform photo-current spectroscopy to obtain the fine infrared absorption spectra^16^. We also performed electrical transport measurements within the same setup (see the “Methods” section). Figure 1b schematically shows the experimental setup of Fourier transform photocurrent spectroscopy. We performed photo-current measurements with 1 V source–drain bias and zero doping to maximum the intrinsic response from the BP channel (see Supplementary Note 1 for details).

**Fig. 2:**
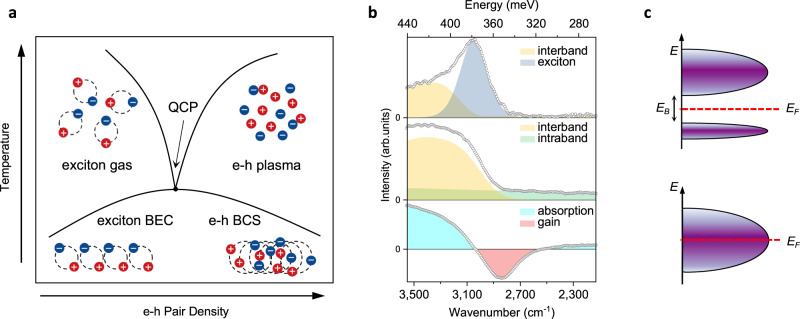
Photo-excited electron–hole system in BP. **a** Schematic phase diagram for correlated electron-hole states in the parameter space of temperature and electron–hole (e–h) pair densities. At low density, the electrons (blue circles) pair with holes (red circles) to form exciton phase. At high density, the electrons and holes exist as Fermi liquids at high temperatures. The boundaries are shown in black lines and cross at the quantum critical point (QCP). At much lower temperatures, the electron–hole system condenses to exciton Bose–Einstein condensation (BEC) and the Bardeen–Cooper–Schrieffer (BCS) states depending on the paring strength. **b** The photocurrent spectra of BP in arbitrary unit (arb. unit). Top panel: under 0.5 W/cm^2^ and at 200 K. The absorption includes an inter-band part and an exciton peak. Middle panel: under 32 W/cm^2^ and at 260 K. The absorption continuously extends to low energy region, indicating an intra-band transition. Bottom panel: under 160 W/cm^2^ and at 5 K. The horizontal lines show the baseline of zero absorption. The turquoise and red regions indicate absorption and gain. Source data are provided as a Source Data file. **c** Schematic of conduction band structure with different interaction strength. With increasing correlation interaction, the conduction band splits into two bands with a Mott gap. *E*_F_ Fermi energy, *E*_B_ exciton binding energy.

**Fig. 3:**
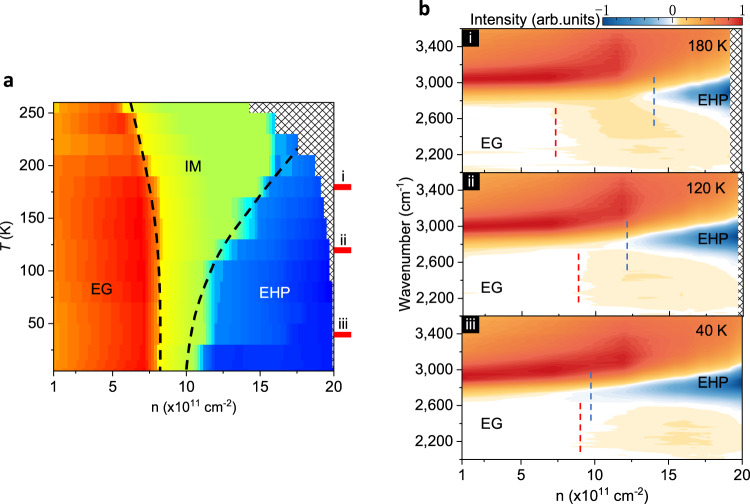
Strong correlated EHP phases in BP. **a** Spectral phase diagram in temperature and e–h pair density space for correlated electron–hole states. In the high-temperature regime, three regions are defined by the characteristics of absorption/gain spectra, where EG, IM, and EHP denote the exciton gas (red), intermediate metal (green), electron–hole plasma (blue) phases, respectively. The boundaries are shown in dashed lines. The shaded area indicates the inaccessible region in the temperature and e–h pair density space. **b** Spectra of phase evolution as a function of e–h pair densities for various *T* marked in **a**, showing the spectral features at different phase transition. The dashed lines indicate the boundaries of EG (red) and EHP (blue) phases. Source data are provided as a Source Data file.

Figure 2b shows typical photocurrent spectra under different photo-excitation and temperatures. For low incident power (e.g. 0.32% *P*, *P* = 160 W/cm^2^), we observe a typical exciton gas phase (EG in Fig. 2a). It features a sharp peak at ~378 meV and continuous absorption at higher energies, which can be assigned as excitonic transition and band-edge absorption (Fig. 2b top panel). The exciton peak with full width at half maximum (FWHM) ~30 meV lies ~9 meV below the band edge, which agrees well with the reported exciton FWHM and binding energy for bulk BP^17^. With increasing excitation power (3.2%*P*–31.6%*P*) and at moderate temperature, we find the absorption spectra distinctively change. The exciton peak disappears and the absorption band extends into the long infrared range (Fig. 2b middle panel). We attribute this cut-off free spectrum to strong intra-band absorption as a result of closing the optical band gap. Notably, the absorption edge corresponding to high-energy interband transition gradually blue-shift (see Fig. 3b). This shift is commonly understood as the “Burstein–Moss shift”. Namely, a degenerate population of carriers blocks optical transition near the band edge. Whereas our results clearly indicate a band renormalization and a metal-to-insulator transition readily occur. We name the forming metallic phase intermediate metal (IM). Under higher excitation power (e.g. 100%*P*) and low temperature, we observe the emergence of a negative dip in the spectrum below the original exciton peak (Fig. 2b bottom panel). The altered spectrum evidences a new phase forms by decreasing the average energy of each electron–hole pair (*E*_eh_), which is distinctively different from simple entropy-driven ionization of excitons. According to semiconductor many-body theory, the exchange and correlation interplays induce density-dependent negative energy into *E*_eh_. Exciton will fully dissociate if these negative components dominate band energy (i.e. *E*_eh_ is smaller than the exciton binding energy)^18^. And the system undergoes a population inversion between the conduction band and valence band because the chemical potential difference of electrons and holes exceeds *E*_eh_. It features the existence of negative absorption (gain) and the formation of strong correlated electron–hole plasma (EHP in Fig. 2a). EHP always results from strongly screened Coulomb interaction, such as in group IV crystals and quantum wells^19–21^. They generally appear in ultra-low temperatures and sufficiently high excitation regimes (carrier density ~10^17^ cm^−3^ obtained by ultrafast pulses of ~10 MW/cm^2^ to GW/cm^2^ power density). In stark contrast, we managed to achieve EHP phases under low power and continuous-wave excitation (full power of 160 W/cm^2^ for the broadband laser covers 2.5–4.8 μm, and the power density threshold of EHP can be down to about 10 W/cm^2^ at 80 K by using a tunable optical parametric oscillator laser ranging from 3.3 to 4.2 μm with pulse repetition rate = 150 kHz and pulse duration = 10 ns).

The access of full electron–hole pair phases permits us to examine the detailed transition between the three phases. We measured a comprehensive phase diagram of the absorption/gain spectrum. Figure 3a summarizes the diagram in temperature and pair density parameter space (see the “Methods” section for estimation of e–h pair density).

### Exciton Mott metal–insulator-transition

We ascribe the phase change from EG to EHP in the diagram as an exciton Mott metal–insulator-transition transition (EMT)^19^ which refers to the dissociation of excitons into strong-correlated electron–hole phases in photo-excited semiconductors. In particular, the aforementioned *E*_eh_ can be obtained by solving the Hamiltonian of electron–hole system:

1 $$H={\sum }_{k}({{\epsilon }}_{k}-{{\epsilon }}_{F})({c}_{k}^{{{\dagger}} }{c}_{k}-{d}_{k}^{{{\dagger}} }{d}_{k})+V{\sum }_{i}{c}_{i}^{{{\dagger}} }{d}_{i}^{{{\dagger}} }{d}_{i}{c}_{i}$$

where *c* and *d* are electron annihilation operators for the conduction and valence bands, *k* is the single-particle momentum, *ϵ*_*k*_ refers to the kinetic energy, *i* labels the real-space site and *V* is the Coulomb interaction. Here we focus on the continuous phase change induced by correlation interplay, and the kinetic energy was chosen as the form of an electron–hole symmetric parabolic band for simplicity. This Hamiltonian can be mapped to a standard repulsive Hubbard model (see the “Methods” section). In this Hubbard-like model, an interaction parameter *U* which relates to electron–hole density modulated Coulomb screening determines the energy spectrum. The ground state of the system is a dense EG, corresponding to half-filled insulating states in the Hubbard model as shown in Fig.2c. The many-body Coulomb correlation results in a split of isolated excitonic states and a continuum which plays as the lower and upper Hubbard bands, respectively. The exciton binding energy equals the difference between these two bands. If the exciton density exceeds a certain threshold under photo-excitation, the screening of Coulomb interaction would be sufficiently strong so that the two band merges into a single one. In this scenario, the Fermi level locates inside the band, excitons dissociate and metallic EHP phases build up.

Figure 3b illustrates the photocurrent spectra at different temperatures. The above EMT process nicely explains the measured spectral evolution. In our experiments, the correlation energy *U* is continuously tuned by electron–hole pair density (through excitation power). The modulation drives the EMT as a quantum phase transition. Our results clearly suggest the EMT transition still takes place approaching zero temperature, evidencing a quantum critical phenomenon^1,4^. In this scenario, the IM phase can be recognized as a quantum critical region.

**Fig. 4:**
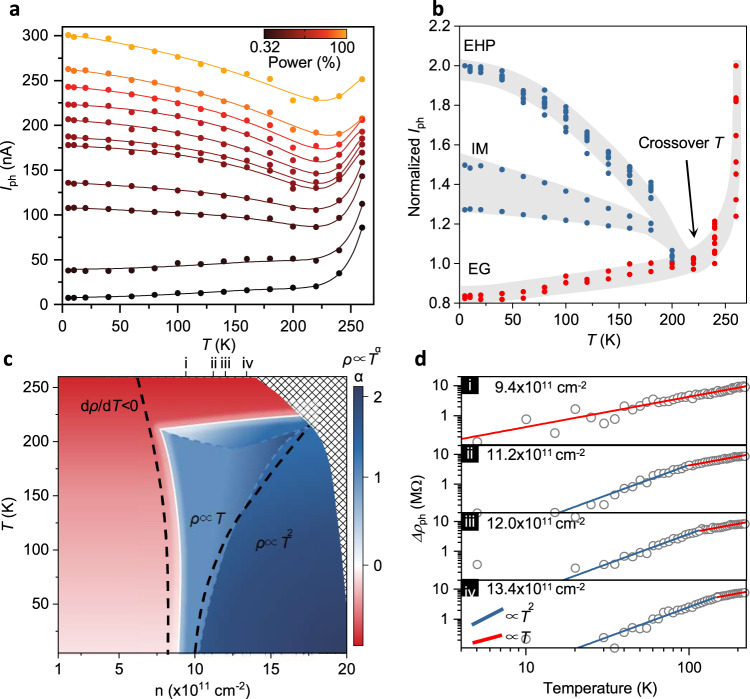
Continuous exciton Mott transition in BP. **a** Temperature dependence of integrated photocurrent at different excitation powers. 100% *P* = 160 W/cm^2^. **b** Photocurrents in **a** normalized to the maximum value at each excitation power. The curves are vertically offset to align the value at EG crossover temperature (220 K, indicated by black arrow) for clarity. The curves display three different branches, showing unconventional metallic properties near the transition boundary. **c** Phase diagram described in terms of the exponent of *T*-dependent resistivity extracted from our transport measurements as a function of *T* and pair density. The diagram clearly features a transition between the insulating phase with a negative coefficient (red) and the metallic phases with a positive coefficient (blue). At the intermediate region, *T*-linear resistivity is observed at low temperatures. Further, into the metal, the resistance starts to develop *T*^2^ behavior. The dashed lines are the boundaries of the IM region in Fig. 3a, showing an agreement between transport and spectral phase transitions. The shaded area indicates the inaccessible region in the temperature and e–h pair density space. **d** Log–log plots of resistivity (circles) versus temperature (below crossover temperature) as well as quadratic (blue) and linear (red) fits for different e–h pair densities near the transition boundary. The blue and red lines are the reference for the *T* and *T*^2^ trends. Source data of all figures are provided as a Source Data file.

To gain a deeper insight about the quantum criticality of EMT, we next discuss transport measurement. In the study of EMT, it is difficult to clearly identify the physical properties of each phase because of the co-existence of optical signals from multiple phases. For example, optical features of multi-species in conventional semi-conductor are too close in energy to be distinctively resolved. We thus performed electrical measurements at temperature and eletron–hole pair density space to elucidate the metal–insulator-transition process. Figure 4a illustrates the temperature dependence of DC current (i.e. photo-current at constant zero optical path difference) up to 260 K at different excitation powers. We normalized the currents by the photocurrents at a crossover temperature (220 K) in Fig.4b to clearly see the temperature evolution. The curves obviously display three different branches, i.e., an unconventional metallic branch between the insulating regime and the ordinary metallic regime.

We further measured detailed differential resistivity to reveal the transport properties. Overall, the resistivity shows a power temperature scaling law: *R* ∝ *T*^α^. We mapped the exponent *α* of the photo-resistivity as a function of electron–hole pair density and temperature in Fig. 4c.

Two types of transport behavior are observed. Below a crossover carrier density (9 × 10^11^ cm^−2^) or above the crossover temperature (220 K), the exponent *α* < 0 (the resistance increases with cooling), featuring an insulator. The temperature dependence obeys a hopping formula, which generally describes low-density carrier transport in disordered 2D semiconductors. At high temperatures, the current exponentially increases with temperature, showing a nearest-neighbor hopping. When the temperature decreases below the crossover point, the current present a much weaker temperature dependence and fits well into a variable range hopping mechanism.

In contrast, the system turns metallic with *α* > 0 above the crossover carrier density and below the crossover temperatures. For sufficiently high carrier density and low temperature, a universal *T*^2^ dependence of resistivity was observed. It characters a conventional Fermi-liquid behavior. The resistance obviously deviates from *T*^2^ dependence at moderate power or high temperature. A *T*-linear dependence was observed near the metal-insulating-transition, and α is higher (but still < 2) near the crossover temperature. It indicates the metallic phases in the vicinity of transition boundaries are different from common metals and analog to a fluctuated non-Fermi liquid^22,23^.

Remarkably, the unconventional metallic region is coincident with the IM region in the spectral phase diagram. The electrical phase diagram is coincident with the optical one. The consistent transport data further explains the IM phase and supports the quantum criticality. In the quantum phase transitions of 2D metals, a ubiquitous feature is so-called strange-metal behavior^1,2,24^. One hallmark of the strange-metal regime is that the electrical resistivity near the phase transition is linearly proportional to the temperature *T*, agreeing well with our measurements as shown in Fig. 4d. (We notice “strange metal” generally refers to a wide variety of unusual metallic behavior at odds with interacting Fermi liquid. Here the term does not correspond to any specific origin.) The *T*-linear resistivity motivates the concept of Planckian dissipation^25^, namely, the temperature is the only scale of scattering rate near quantum criticality. And the divergence of scattering length (no length scale of scattering) is an indication of quantum criticality^26^.

Based on the above observation, a quantum critical point can be also expected when further cooling to near absolute zero. And finally, we comment on the rich correlation physics around the quantum critical point^27,28^. Firstly, for Fermionic systems, fermion pairs condensate at low temperatures. It may result in superconductivity and superfluidity, known as Bardeen–Cooper–Schrieffer states (BCS) and Bose–Einstein condensation (BEC), respectively. The nature of this condensate can be tuned by varying the pairing strength^29^. In our system, the electron–hole pairing strength can be dynamically and continuously adjusted. We theoretically predicted the system allows access to two distinct regimes of exciton condensation and a BCS-BEC crossover at the quantum critical point (see Supplementary Note 2). In addition, at the many-body level, there will be strong correlations and quantum fluctuations that are beyond the theoretical frameworks in the BCS and BEC limits. For example, the normal state at finite temperature has been proposed to describe the aforementioned strange metal phase^30^. The preformed pairs are believed to be the course of the pseudogap in cuprates. However, less is known for the transport behavior of the BCS–BEC crossover at elevated temperatures. And our finding of general *T*-linear resistivity may help explain the universal linear resistivity behavior not only in the pseudogap phase of the cuprates^31,32^, but also in broadly correlated materials^24,33,34^. Experimentally, the large carrier density at the quantum critical point indicates the BEC–BCS crossover may be accessed at about 0.2–2 K^30^. Future exploration toward BCS and BEC states with cryogenic cooling down to the sub-Kelvin range could elucidate the rich exciton condensation physics.

## Methods

### Device fabrication

The all-2D multilayer heterostructure was stacked using a dry transfer technique. The whole fabrication process was carried out in an argon-filled glove box with both oxygen and water concentrations well below 0.1 ppm. The hBN, graphene, and BP flakes were first exfoliated with blue tapes and sequentially picked up using polydimethylsiloxane (PDMS) held on a glass slide. The quality and thickness of samples were identified via optical microscopy by comparing optical contrast with samples whose thickness was confirmed using atomic force microscopy (AFM, Nanonics MultiView 4000). The samples on PDMS were released using the micro-positioning stage (Zolix NFP-6561) at room temperature in the following order: (1) few-layer hBN flake on SiO_2_/Si substrate (285 nm SiO_2_); (2) few-layer graphene ribbons on hBN as drain and source electrodes; (3) ~10 nm BP flake bridging two graphene ribbons; (4) ~20 nm hBN flake covering the entire BP as top dielectric, and (5) monolayer graphene as a top-gate electrode. Then 5 nm Cr/35 nm Au contacts were patterned with standard electron-beam lithography, electron-beam evaporation, and lift-off processes.

### Fourier-transform photocurrent spectroscopy

The absorption/gain spectra were performed by a homemade Fourier transform infrared (FTIR) photocurrent spectrometer system schematized in Supplementary Fig. 3. In brief, the device was placed in a vacuum environment in a closed-cycle optical cryostat (Montana S50) where temperatures ranging between 5 and 260 K. A broadband mid-IR supercontinuum laser (LEUKOS Electro MIR 4.8, 0.8–4.8 μm) light source was modulated by an FTIR spectrometer (BRUKER Invenio-R): The incident laser beam was then split through the beamsplitter. The transmitted one and the reflected one struck the stationary mirror and the movable mirror, respectively. Then, two beams of light were reflected by the mirrors and recombined with each other at the beamsplitter. The constructive or destructive interference occurred because of the optical path difference induced by the movable mirrors. As a result, the laser became a modulated wave after traveling through the Michelson interferometer. We used a ×40/0.5NA reflective microscope objective (Thorlabs) to focus the modulated beam on the device. A series of IR neutral density filters (Edmund 2–14 μm) with optical density ranging from 0.3 to 2.5 was used to control the illumination power. An infrared long pass (Edmund 2.52–4.8 μm) filter was employed to block the photon beyond our interested spectrum range. A low-noise current preamplifier (Stanford SR570) was used to collect the photocurrent and supply source–drain voltage. The amplified photocurrent signal was fed back to the FTIR spectrometer and was recorded while the incident laser being modulated. The photocurrent interferogram obtained in this way is inversely Fourier-transformed by the FTIR to the frequency domain. The positive and negative photocurrents are proportional to optical absorption and gain, respectively (See Supplementary Note 1 for details).

### Photocurrent and transport measurement

The electrical measurements were performed on the same cryogenic station and light source as the Fourier-transform photocurrent spectroscopy (see Supplementary Note 3 for details). In these experiments, the Michelson interferometer was fixed at zero optical path. The DC photocurrent was directly read by a semiconductor analyzer (PDA FS-pro). The photo-conductance was measured by standard low-frequency lock-in scheme. A DC bias with a small AC excitation voltage (1 mV) at frequency of 11 Hz was applied to the sample by a source (Keithley 6221). Corresponding current flowing through the sample was then measured by a lock-in amplifier (SSI OE1022).

### Estimation of e–h pair density

The electron–hole pair density values are given in the figure caption of Figs. 3a, 4c have been extracted by comparing the photocurrent and gate-dependent dark current (for example, see Supplementary Note 1)^21,35^. We also calibrated the density from the excitation pulse energy flux and estimated the absorption coefficient at low density^36^, and by a line-shape fit for the highest density^37^.

### Exciton Hubbard-like model

We adapted the method in ref. 38 to transform the Hamiltonian to a standard Hubbard model. The semiconductor in our case can be roughly described by the Hamiltonian of Eq. (1). We will not be concerned with the details of the band dispersion *ϵ*_*k*_ except for *ϵ*_*k*_ ≥ 0, and for analytical convenience, we will assume that the density of states *N*_0_ is a constant over the bandwidth *W*, unless it is pointed out otherwise. The Fermi energy *ϵ*_F_, measured from the bottom of the conduction band, is negative for an unperturbed semiconductor (whence the direct gap *D* between the valence and conduction bands is −2*ϵ*_F_). In the above Hamiltonian, we assumed the valence and conduction bands are exactly particle–hole symmetric. This assumption makes the discussion and calculation more convenient without the loss of generality. We have also ignored the spin degrees of freedom, which is irrelevant in semiconductors. Finally, the last term in the Hamiltonian *H* describes the local Coulomb repulsion between electrons in the two bands. We ignored the intra-band electron interaction since it is irrelevant to the low density of electrons (holes) in the conduction (valence) bands. In the quasi-stationary state under pumping, electrons (holes) are pumped into the conduction (valence) band, and this may be described qualitatively by a positive Fermi energy *ϵ*_F_ > 0 (if the interaction is ignored).

The assumed perfect particle–hole symmetry enables us to map the system into an effective one-band model with artificial spin-1/2 degrees of freedom,

2 $$H\to {\sum }_{k,\sigma }({{\epsilon }}_{k}-{{\epsilon }}_{F}){c}_{k\sigma }^{{{\dagger}} }{c}_{k\sigma }-U{\sum }_{i}{c}_{i\uparrow }^{{{\dagger}} }{c}_{i\downarrow }^{{{\dagger}} }{c}_{i\downarrow }{c}_{i\uparrow }$$

where *σ* = ↑,↓, the fermion annihilation operator *c*_↑_ (*c*_↓_) corresponds to *c* (*d*^†^) in the above semiconductor model, and the interaction becomes attractive in the new representation.

## Supplementary information

Supplementary Information

## Data Availability

The data that support Figs. 1–4 can be found in the Source Data, and the data that support the other findings of this study are available from the corresponding author upon reasonable request. Source data are provided with this paper.

## Source data

Source Data
