# Supplementary material for: Quantum criticality of excitonic Mott metal-insulator transitions in black phosphorus
Source: Nat Commun. 2022 Dec 17;13:7797. doi: 10.1038/s41467-022-35567-w (PMC9759515; doi:10.1038/s41467-022-35567-w)
Supplement: Supplementary file 1 — Supplementary Information [file 41467_2022_35567_MOESM1_ESM.pdf]

Supplementary Information for

Quantum Criticality of Excitonic Mott Metal-insulator Transitions in Black Phosphorus

Binjie Zheng[1,3] , Junzhuan Wang[1,3], Qianghua Wang[2], Xin Su[1], Tianye Huang[1],

Songlin Li[1], Fengqiu Wang[1], Yi Shi[1] and Xiaomu Wang[1*]

1 School of Electronic Science and Engineering, Nanjing University, Nanjing 210093, China

2 School of Physics, Nanjing University, Nanjing 210093, China

3 These authors contributed equally to this work

*Corresponding authors: xiaomu.wang@nju.edu.cn

Table of contents

Supplementary Note 1 | Characterization the photo-response of BP channel………………………3

Supplementary Note 2 | Crossover from BEC to BCS at quantum critical point ……………….….5

Supplementary Note 3 | Experimental setup for the Fourier Transform Photocurrent Spectroscopy and Transport Measurement………..…………10

Supplementary Note 1. Characterization the photo-response of BP channel

We characterize the photo-response of BP channel to validate the Fourier transform photo-current spectroscopy. **Supplementary Fig. 1a** shows photocurrent as a function of source-drain bias and back gate voltage. **Supplementary Fig. 1b** further summarizes the photo-responses with constant doping and bias voltage. The positive photocurrent implies it results from a photovoltaic-like mechanism, where electron-hole pairs are formed under global light excitation and then separated by electric fields. The photovoltaic current can be estimated from $J = qn^{*}\mu E$, where q is the electronic charge, n^{*} is the photo-generated electron-hole pair density, E is the electric field and μ is the carrier mobility of our device. In general, the n^{*} value is almost unchanged with moderate doping. As a result, the photocurrent only slightly increases with doping dependent (p-type) carrier mobility, and the photo-to-dark current ratio is maximized at charge neutral point. In addition, the IV curve indicates the contacts are asymmetric for this specific device: the source electrode is ohmic and the drain electrode is Schottky. In this scenario, externally applied electric field mainly separates photogenerated carrier for forward bias, and the built-in field dominates for reverse bias. The whole channel (Only contact region) responses in the former (later) case. The spatial photocurrent mapping shown in the **Supplementary Fig. 1c** verifies the photocurrent produced uniformly along the BP channel. Accordingly, to best reflect BP’s optical properties, we selected forward Source-Drain bias and zero doping gate voltage for our studies.

Notably, in this circumstance, we are able to measure the photocurrent as a function of wavelength and deduce the infrared spectrum by Fourier transform photocurrent spectroscopy. The key point of the method is the absorption at each wavelength is proportional to the photo-current. This is true (in the mid wave infrared range) because the photovoltaic current is the product of carrier mobility, absorption rate and incident power density. The spectrum we measured thus scale with the absorption as the mobility and incident power density are constants.

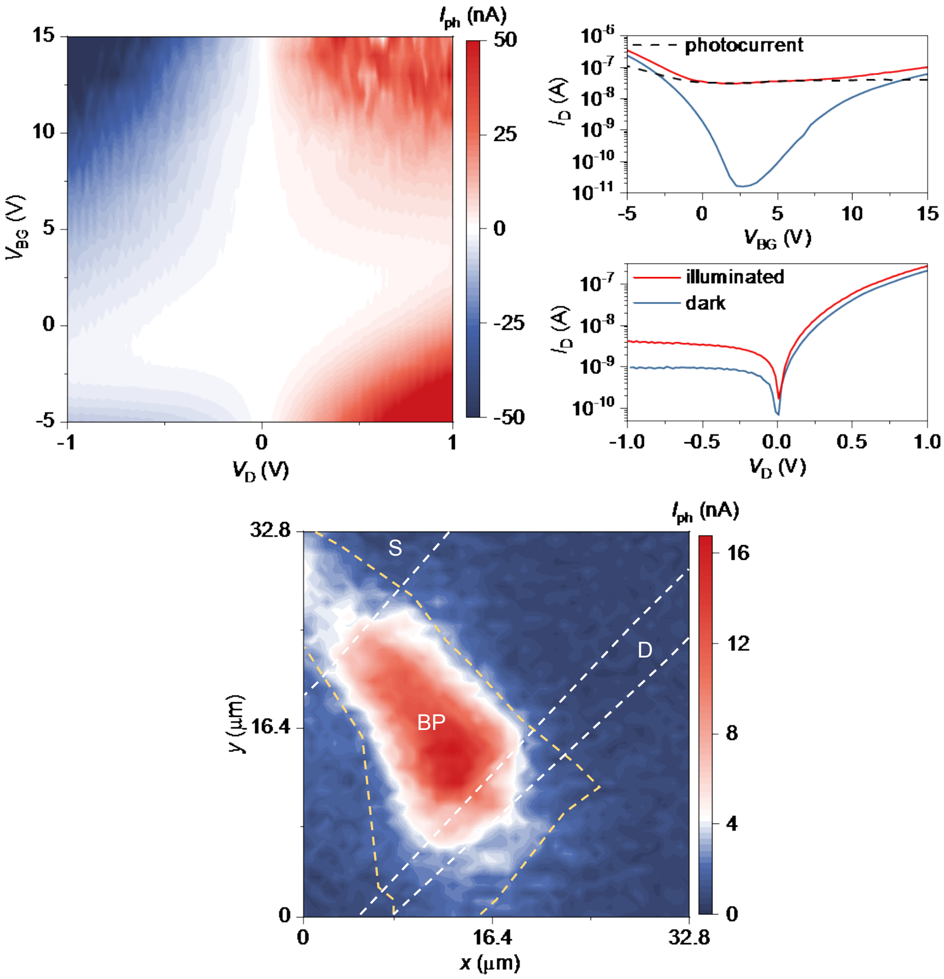

Supplementary Fig. 1 | Characterization the photo-response of BP channel. a, Photocurrent (I_{ph}) mapping as a function of the V_D and V_{BG}. **b**, Photo-responses with constant doping (top panel, V_D = 1 V) and bias voltage (bottom panel, V_{BG} = 0 V). **c**, Spatial photocurrent mapping of the BP device in **b**. V_D =1 V, V_{BG} =0 V. The yellow and white dashed lines represent the edges of BP and graphene electrodes, respectively.

Moreover, we attributed the negative photocurrent to gain features (or equally, population inversion). In stark contrast to the positive photocurrent where light excites extra carriers into BP channel, the negative photocurrent indicates (externally) injected carriers in the channel are reduced. (Noting in the wavelength range with negative photocurrent, the photo-current mechanism should be unchanged. Because this wavelength range varies with temperature and incident power, and most spectra

still present positive photocurrent in the same BP channel. And this wavelength range is also of positive photocurrent under low light excitation. It evidences no special wavelength dependent and different photo-current mechanism.) As illustrated in **Supplementary Fig. 2**, the most reasonable explanation of negative photocurrent is light stimulates carriers decay (including but not limited by emission) at a rate higher than the photo-carrier generation rate; or in other words, population inversion occurs.

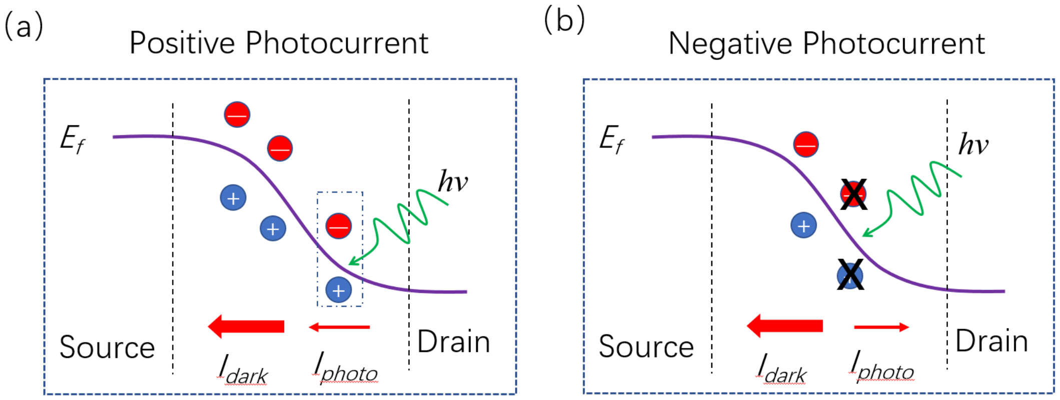

Supplementary Fig. 2 | Schematics of photocurrent mechanism. a. The positive photocurrent results from a photovoltaic-like mechanism, where excessive electron-hole pairs are excited under light excitation and then separated by electric fields. **b.** The negative photocurrent results from electron-hole pairs are inversely consumed by light excitation, suggesting the channel undergoes population inversion where electron-hole recombination rate is higher than its generation rate, or equally photo-gain exists.

Supplementary Note 2. Crossover from BEC to BCS at quantum critical point

The Hubbard-like Hamiltonian in the maintext is a typical Bardeen-Cooper-Schrieffer (BCS) Hamiltonian describing simple s-wave superconductors[1], except that here the pairing interaction comes from direct Coulomb interaction (between particles and holes in the semi-conductor language), rather than the effective interaction mediated by phonons. The behavior of the BCS Hamiltonian is well understood, and can be qualitatively captured by mean field treatment in all of the interaction range in our concern[2]. The pairing gap Δ is determined by the BCS gap equation

$$\frac{1}{\lambda}=\int_{-\epsilon_F}^{W-\epsilon_F} d\epsilon \frac{1}{2E}\tanh\frac{\beta E}{2},$$

Here $\lambda = N_0 U$ is the coupling constant, and $E=\sqrt{\epsilon^2+\Delta^2}$ is the quasi-particle energy in the paired state, given its energy $\epsilon=\epsilon_k-\epsilon_F$ in the normal state. Quasiparticle excitations are gapped by the minimum of E, which is just Δ if $\epsilon_F > 0$, but would be given by $\sqrt{\epsilon_F^2+\Delta^2}$ if $\epsilon_F<0$. The particle density (per site per spin) is given by

$$n = N_0\int_{-\epsilon_F}^{W-\epsilon_F} d\epsilon\,\frac{1}{2}\left(1-\frac{\epsilon}{E}\tanh\frac{\beta E}{2}\right).$$

For weak coupling $\Delta\to 0,$ this equation is essentially equivalent to the integration over the standard Fermi function for the normal state, hence the particle density would be unchanged in the paired state. In the opposite limit, if Δ is large, the above density equation is essentially different to that in the normal state, and the Fermi energy must be adjusted (automatically by the system) to match the given particle density. We shall discuss these limits in more details below. We remark that the electron pairing in the BCS theory maps to exciton condensation in the semi-conductor, as seen from the mapping between the order parameters $\langle c_\downarrow c_\uparrow\rangle=\langle d^\dagger c\rangle$. We will switch between these pictures in due course.

Weak coupling BCS limit: First we consider the case of strong pumping, such that the particles pumped into the conduction band is relatively high, while the interaction is

relatively weak. For example, we take $\epsilon_F > 0$, and $U \ll \epsilon_F$. This case is well described by the weak-coupling BCS theory. Solving the BCS equation in the zero temperature limit, one obtains

$$\Delta \sim 2\sqrt{\epsilon_F(W-\epsilon_F)}e^{-\frac{1}{N_F U}}.$$

Below half-filling ($\epsilon_F < \frac{W}{2}$), the gap increases with ϵ_F (or the pumping flux) for two reasons. First, the pre-factor in Δ increases. This can be traced back to the fact that more particles participate pairing. On the other hand, for a three-dimensional parabolic dispersion ϵ_k, the density of states at the Fermi level actually increases as $N_F \propto \sqrt{\epsilon_F}$, and this enhances the BCS coupling constant $\lambda = N_F U$ in place of $\lambda = N_0 U$, as ϵ_F increases. The low-energy single-particle density of states in the `superconducting' state is given by

$$\rho(\omega) \sim N_0 \frac{|\omega|}{\sqrt{\omega^2-\Delta^2}}, \qquad \Delta \leq |\omega| \ll \epsilon_F,$$

where ω is the quasiparticle energy. It is particle-hole symmetric near the Fermi level, and has well-defined coherence peaks (square-root singularity) at the gap edge $\omega = \pm\Delta$. According to the behavior of the single-particle gap versus the Fermi energy discussed above, we expect an increase (or a blue shift) of the light absorption gap (which is 2Δ) as the pumping flux increase. The length scale of the Cooper pair is given by the coherence length $\xi \sim v_F/\Delta$, where v_F is the Fermi velocity. So in the weak coupling limit $\Delta \ll \epsilon_F$, the Cooper pairs are composed of loosely bounded electrons, and overlaps over a large coherence length compared to the Fermi wavelength.

<u>BEC limit</u>: If the pumping flux is weak, such that the density of electrons pumped into the conduction band is low, we may reach the limit $U \gg |\epsilon_F|$. This is the so-called BEC limit, with Bose-Einstein condensation of tightly bound dimers of electrons at low temperatures. Qualitatively, if $\epsilon_F \leq 0$, then v_F is either zero or undefined, such that $\xi = v_F/\Delta \to 0$, meaning the electrons are tightly bound within Cooper pairs. In this limit, the fermions first form tightly bound dimers of fermions, and the dimers may condense subsequently. Since our pump-probe experiment is only sensitive to

processes composed of single-particle excitations, we will not be concerned with the condensation of dimers. The formation of fermion pairs can still be reliably captured by the BCS theory. However, since the pairing is strong, it requires adjustment of the Fermi energy in order to match the given particle density. Let us assume that we are deep in the BEC limit such that $\epsilon_F < 0$. In the zero temperature limit, the gap equation yields

$$\Delta_m = \sqrt{\epsilon_F^2 + \Delta^2} \sim We^{-\frac{2}{\lambda}}.$$

Here Δ_m denotes the minimal gap for quasiparticle excitation. The density equation yields

$$n \sim \frac{1}{2} N_0 \sqrt{\epsilon_F^2 + \Delta^2} = \frac{1}{2} N_0 \Delta_m.$$

This implies that the excitation gap increases with particle density also in the BEC limit. The strict linear relation between n and $\Delta_m$is specific to our assumption of constant normal state density of states N_0, but we the qualitative behavior is unchanged for more realistic energy dependence in N_0.

The low energy quasiparticle density of states is now given by

$$\rho(\omega) = \frac{N_0}{2}\left(\frac{|\omega|}{\sqrt{\omega^2 - \Delta^2}} + \frac{\omega}{|\omega|}\right)\theta(|\omega| - \Delta_m).$$

Notice that the excitation spectrum is no longer symmetric about zero energy, in contrast to the case in the weak coupling limit. The spectral weight in the negative energy side is much smaller and vanishes quickly, and in fact it integrates to the particle density n. Moreover, since the density of states begins to show up only after $\omega^2 \geq \Delta^2 + \epsilon_F^2$, there is no singularity at the gap edge in $\rho(\omega)$, except for a finite jump from zero to finite density of states as $|\omega|$ increases beyond Δ_m.

<u>BCS-BEC crossover</u>: The weak coupling limit can be reliably treated by the traditional BCS theory, and in the BEC limit, the BCS treatment is also reliable for the gap opening and single-particle excitations. The subsequent dimer condensation can be treated in the framework of dilute weakly interacting (composite) bosons, and at

this stage can also be classified as a weak coupling problem. The physics becomes more involved and interesting in the crossover regime between the BCS and BEC limits. To classify the interaction strength, it is more convenient to resort to the scattering length a in a two-body scattering problem, in the continuum limit[2]. A large and negative $1/k_c a$ ($1/k_c$ is an atomic length scale) corresponds to weak attraction. This is the BCS limit. If $1/k_c a$ is large and positive, the attraction is strong enough to bind the two fermions into a bound state already at the two-particle level. This is the BEC limit. The intermediate situation is $\frac{1}{k_c|a|} \sim 1$. When the attraction reaches the so-called unitary limit, $\frac{1}{k_c a} \sim 0$ from the negative (positive) side, the particles form a loose resonance (a loose bound state) at the two-particle level. The dynamical and continuous tunability of electro-hole coupling strength in a solid-state device provides a new opportunity to study the phenomenology of exciton condensates at various pairing strengths, which may lead to deeper understanding of unconventional superconductivity.

Supplementary Note 3. Experimental Setup for the Fourier Transform Photocurrent Spectroscopy and Transport Measurement

The absorption/gain spectra were performed by homemade Fourier transform infrared (FTIR) photocurrent spectrometer system schematized in **Supplementary Fig. 3.** Briefly, a broadband laser was modulated by a Michelson interferometer and focused on the sample (see Method for details). Under this illumination, electron-hole pairs generate in the BP channel and result in a photo-current proportional to the infrared absorption. During the measurement, incident light containing all wavelength reached the BP channel simultaneously. We then recorded the photocurrent signal as a function of the interferometer delay. And the absorption spectrum covering full spectral components can be derived from inverse Fourier transform of these temporal records. This methodology permits obtaining comprehensive absorption covering the entire band edge with ultra-high resolution.

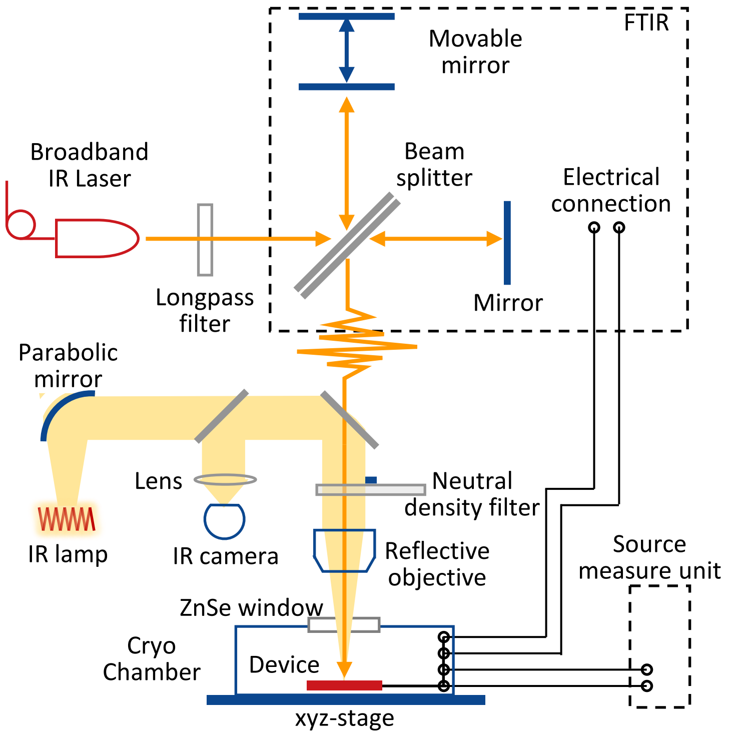

Supplementary Fig. 3 | Schematic of FTIR photocurrent spectrometer system.

The electrical measurements were performed on the same cryogenic station and light source as the Fourier-transform photocurrent spectroscopy. In these experiments, the

Michelson interferometer was fixed at zero optical path. The DC photocurrent (Fig. 4 a and b) was directly read by a semiconductor analyzer (PDA FS-pro). The photo-conductance (Fig. 4c and d) was measured by standard low-frequency lock-in scheme. A DC bias with a small AC excitation voltage (1 mV) at frequency of 11 Hz was applied to the sample by a source (Keithley 6221). Corresponding current flowing through the sample was then measured by a lock-in amplifier (SSI OE1022).

Here we used 2-wire setup. This is reasonable because for the phases we quantitatively concerned (i.e. the metallic phases IM and EHP), the contact is nearly transparency. Supplementary Fig .4 shows the differential conductivity *dI/dV* versus bias voltage of our samples at typical IM phase and EG phase at 200K. Obviously, the *I-V* curve for the IM phase is linear. The differential conductivity stays flat for all applied bias voltage. We thus used constant 100mV DC bias voltage for the transport measurement. On the contrary, the I-V characteristic becomes nonlinear for the EG phase where the sample is very insulating. Fortunately, we do not need a clearly defined scaling factor for the EG phase, thus the contact effect does not affect our result.

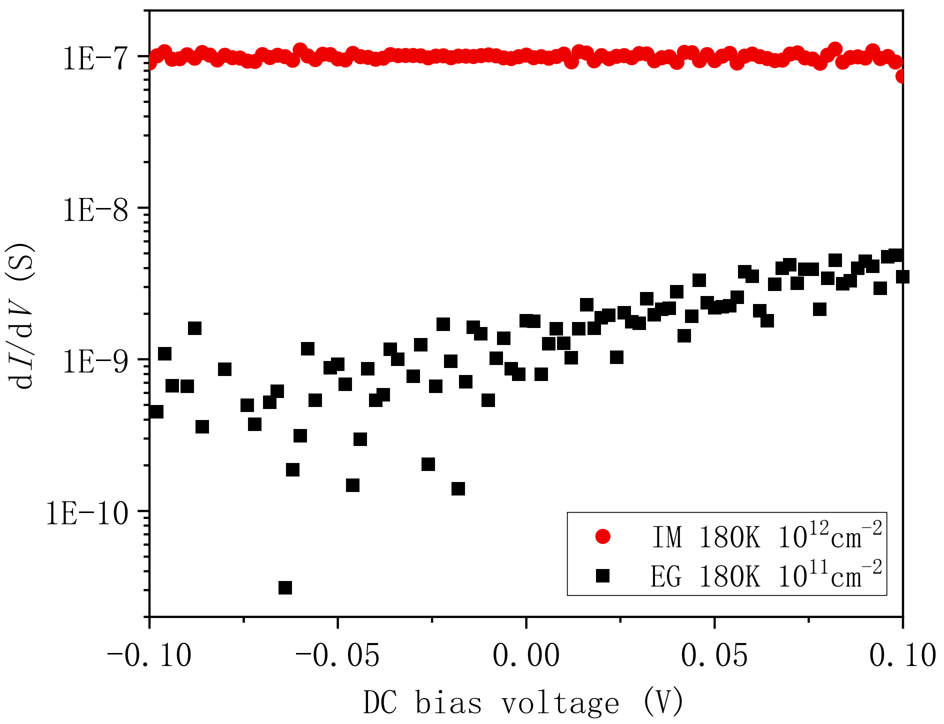

Supplementary Fig. 4 | Contact effect of 2-wire Transport measurement. Bias voltage (*V*) dependence of the differential conductance *dI/dV* at 200K under varying excitation power. The ac modulation voltage is 1mV. The differential conductance is independent of *V* for the whole range.

Supplementary Reference

[1] J. R. Schrieffer. Theory of Superconductivity. Benjamin edtion. (1964)

[2] A. J. Leggett & R. Przystawa. Modern Trends in the Theory of Condensed Matter. Springer Verlag, Berlin, (1980)
